# Low-cost 3D-printed temporal bone model for ear surgery training

**DOI:** 10.1016/j.bjorl.2026.101936

**Published:** 2026-10-31

**Authors:** Stefano Tincani, Carlos Takahiro Chone, Rebecca Christina Kathleen Maunsell

**Affiliations:** a Universidade Estadual de Campinas, Campinas, SP, Brazil; b Universidade Estadual de Campinas, Otolaryngology/Ophthalmology Department, Campinas, SP, Brazil

**Keywords:** Printing, three-dimensional, Temporal bone, Internship and residency, Ear surgery, Models, educational

## Abstract

**Objectives:**

Our objective was to develop a low-cost 3D-printed temporal bone model for ear surgery training, analyze all costs involved in its production, and assess its quality.

**Methods:**

This article provides a thorough overview of the development process for a 3D-printed temporal bone, detailing both the initial investment and production costs involved. Additionally, an online questionnaire was conducted to evaluate the model’s quality, focusing on anatomical accuracy and the dissection experience.

**Results:**

Training otolaryngology residents and fellows rated all aspects of the model positively. The anatomy section received high ratings, particularly for bony structures, and the utility for training achieved a perfect score. A cost analysis indicates that our model has the lowest cost reported in the literature to date.

**Conclusion:**

Our innovative model not only offers low costs but also achieves outstanding quality ratings. By effectively addressing the critical shortage of cadaveric temporal bones, this solution improves ear surgery training at teaching centers, equipping the next generation of surgeons with the necessary skills for success. Investing in this model is not just practical; it represents a significant advancement in medical education and training.

**Level of Evidence:**

5.

## Highlights

• A very low-cost temporal bone model for ear surgery training was achieved and described.
• With US$ 455.65 initial investment was made, and the materials cost per unit was US$ 3.93.
• After dissection, the model was highly rated by residents and otology fellows.

## Introduction

The complex anatomy of the temporal bone demands detailed knowledge and repetitive training, traditionally performed in laboratories specifically equipped for temporal bone dissection to build learners' practical experience. Cadaveric specimens have traditionally been the standard for temporal bone surgery training; however, their decreasing availability and rising costs have driven demand for alternative options.^1^, ^2^, ^3^

While cadaveric specimens remain the gold standard, alternatives such as virtual reality training and 3D printing are increasingly viable options, particularly for beginners in ear surgery.^4^^,^^5^ Several authors have demonstrated 3D-printed models for ear surgery training. Since the first models were described, 3D printers have improved in both cost and quality.^6^ Lower costs allow more frequent training, supporting progress along the student's learning curve. Commercially available models currently range from USD 155.00 to USD 285.00,^7^^,^^8^ whereas a cadaveric specimen can cost approximately USD 2,000.00. The least costly alternative is to 3D-print specimens in-house.^9^

Cost is of particular concern in developing countries, where resources may be limited. To address this, we developed and 3D-printed a custom low-cost temporal bone model. The aim of this study was to describe the development, manufacturing workflow, production cost, and preliminary educational evaluation of an ultra-low-cost 3D-printed temporal bone model.

## Methods

### 3D object development

A temporal bone model based on Med-El's OpenEar library^10^ was developed. The OpenEar library is an open-source archive of eight temporal bones reconstructed in three dimensions using cone-beam computed tomography and micro-slicing. The model used is identified as “Theta” in the OpenEar library. Using Blender,^11^ a free and open-source 3D modeling program, and Lychee Slicer,^12^ the software that generates files for the 3D printer, we modified the original 3D object. Blender was used to close gaps in the Fallopian canal of the original bone model to make the canal airtight. These gaps may represent true dehiscences in the original bone or, alternatively, failure of the OpenEar reconstruction methods to resolve the thin walls of this canal. In addition, a breakaway syringe inlet was added at the stylomastoid foramen. The purpose of these modifications was to allow silicone to be injected into the Fallopian canal to represent the facial nerve in the teaching model.

To achieve mobility of the ossicular chain, the short process of the incus was detached from the temporal bone by creating a thin gap in the bone. In the slicer program, small supports representing ligaments were added in their normal anatomical positions (tensor tympani and stapedius muscles, malleus ligaments). An additional orifice was placed at the mastoid tip to allow complete drainage of residual resin from the mastoid air cell system.

The model was printed using Bene5, a consumer-grade Masked Stereolithography (MSLA) 3D printer, also known as a resin printer, manufactured by NOVA3D.

After printing, the object was washed with 99% Isopropyl Alcohol (IPA). The outer surfaces were scrubbed with a toothbrush, and the inner cavities were rinsed repeatedly using a 20 mL syringe. The syringe was inserted through an orifice created by 3D modeling on the medial side of the mastoid tip and through the internal and external acoustic meatus. Once the IPA had fully dried, a commercially available platinum-cure silicone (Shore hardness 20A) was injected through the Fallopian canal until it emerged from the internal acoustic meatus. After a silicone curing time of 24-h, to achieve final curing, the resin was exposed to direct sunlight for 5-min while the model was submerged in water in a small plastic bowl. This step helped dissolve any residual resin trapped inside the model, which would otherwise leak and alter its external appearance. After a further drying phase, the sigmoid sinus, carotid canal, and cranial base were painted using blue and pink markers (Faber-Castell Felt Tip Pen with Soft Brush Nib), available in ordinary stationery shops. These long, soft-tipped pens facilitated access to the carotid canal, the most difficult of the painted structures to reach (Fig. 1, Fig. 2).

**Fig. 1:**
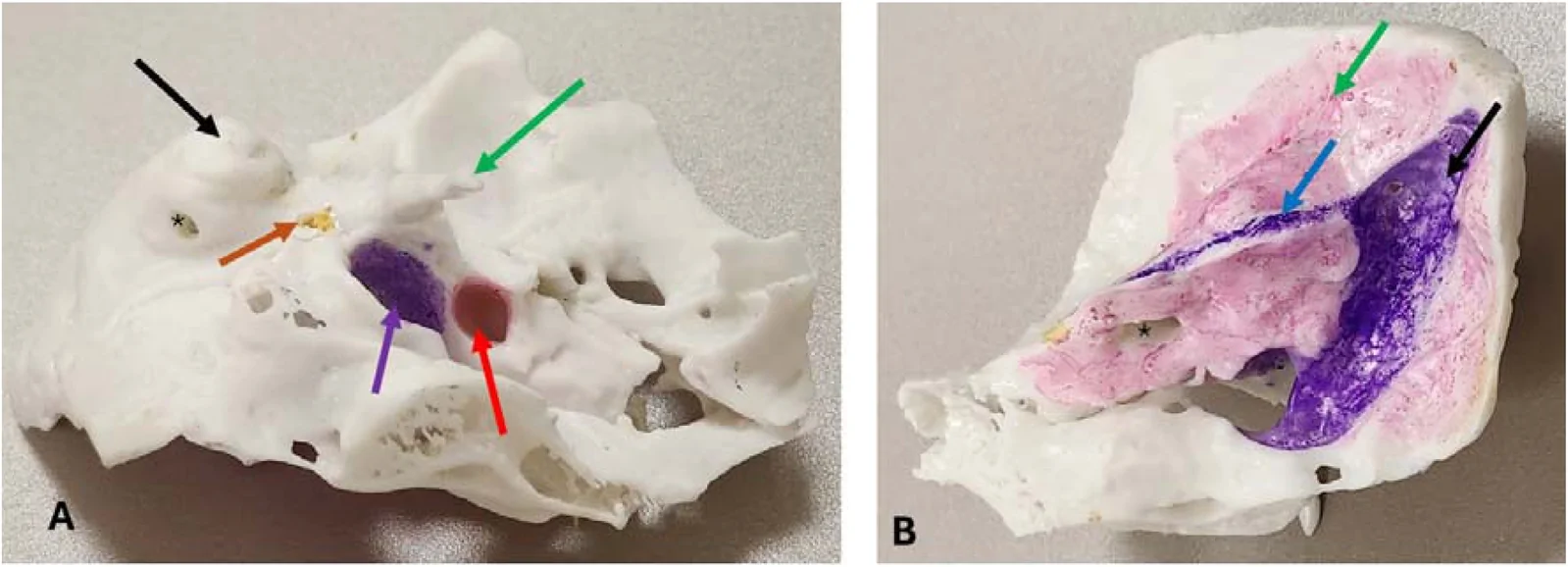
Appearance of the model ready for dissection. (A) Inferior view. From left to right: Black arrow, Mastoid tip; Orange arrow, Stylomastoid foramen (facial nerve); Purple arrow, Jugular foramen; Red arrow, Carotid foramen; Green arrow, Styloid process. Asterisk, Hole for resin drainage from the mastoid cells. (B) Posterosuperior view. Blue arrow, Superior petrosal sinus; Black arrow, Sigmoid sinus; Green arrow, Dura mater; Asterisk, Internal acoustic meatus.

**Fig. 2:**
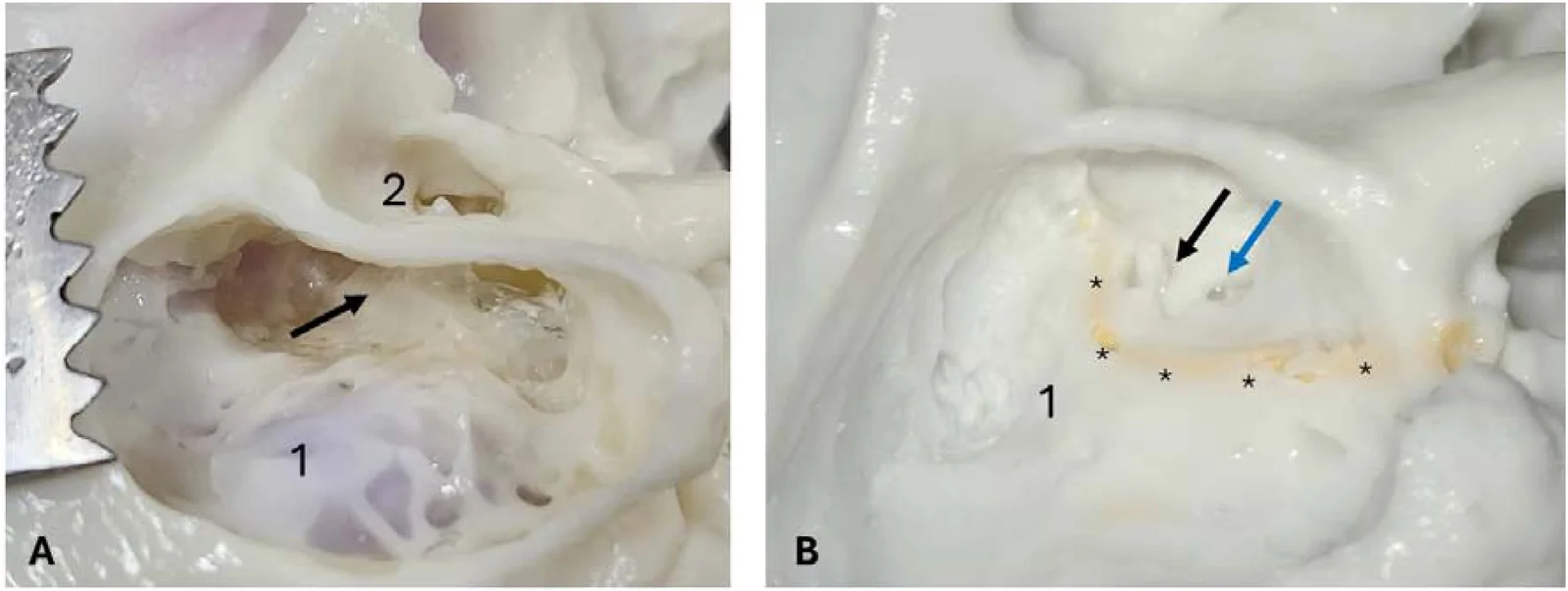
Appearance during dissection. (A) Mastoidectomy with posterior tympanotomy. 1. Sigmoid sinus coloring visible through the transparent wall; 2. External acoustic meatus and malleus; Arrow: Lateral semicircular canal. (B) Canal-wall-down procedure with the ossicular chain removed. 1. Lateral semicircular canal; Black arrow: Oval window with stapes; Blue arrow: Round window; Asterisks: Fallopian canal containing yellow silicone representing the facial nerve.

### Cost analysis

All steps in the production of the model were documented and all costs recorded. For consumables, the prices actually paid at the time of purchase were used for calculations. All values are reported in US dollars; when necessary, conversions were performed using the exchange rate in effect on the day of purchase. The per-unit cost of each material was calculated directly or estimated when direct measurement was not feasible. After the initial investment and per-unit costs were established, total cost was projected as a function of the number of units produced.

Consumables per unit were quantified, including resin, isopropyl alcohol, and electricity, as well as single-use items such as gloves. Materials that were difficult to quantify precisely, or that varied between units, were deliberately overestimated. For example, every printed unit retains excess resin on its surface, which must be washed off with isopropyl alcohol; this alcohol can be reused several times until saturated. The resin volume per unit was based on the volume of the three-dimensional object estimated by the slicer software, plus an additional 50% to account for the resin washed away during cleaning. The same volume was assumed for isopropyl alcohol.

### Dissection protocol

Otolaryngology residents and otology fellows at our institution were invited to perform experimental dissection on the model to assess its perceived quality and usefulness. Residents were asked to perform a simple mastoidectomy exploring the surgical limits, followed by exposure of the ossicular chain. They were then required to perform either an exploration of the facial nerve or a canal-wall-down mastoidectomy. All procedures were done in our institution’s dissection laboratory, with a surgical microscope, temporal bone holder, surgical drill, irrigation and suction.

### Questionnaire

After dissection, participants completed an online questionnaire that assessed their previous experience, their impressions of the anatomical structures encountered, and the surgical steps they were able to complete. Previous experience was quantified by the number of years of otolaryngology practice (including residency and/or otology fellowship) and by previous experience with ear surgery.

Participant perceptions of the model were assessed using a Likert scale, a method commonly used in initial evaluations of similar teaching models.^13^^,^^14^ Participants answered the following questions on a scale from 1 (worst) to 5 (best):

• Based on your current knowledge and experience, how do you evaluate the quality and detail of the bone anatomy encountered?
• Based on your current knowledge and experience, how do you evaluate the soft tissue anatomy encountered: sigmoid sinus, dura mater, and facial nerve?
• Based on your current knowledge and experience, how do you evaluate the bone dissection experience (tactile, visual, and auditory feedback).
• How do you rate the utility of this temporal bone model for ear surgery training?
• How do you perceive the safety of the synthetic model, when compared to a human temporal bone (fresh or fixed in formalin)?

### Statistical analysis

Mean scores were calculated for all responses and stratified by participant experience. Spearman's rank correlation coefficient was used to assess whether responses differed according to participant experience.

## Results

The cost per unit produced was USD 3.93, excluding the initial investment of USD 455.65. The full cost analysis is summarized in Table 1. The initial investment comprised all permanent or long-lasting items used for production or workflow.

**Table 1:** Cost analysis of production.

Initial investment		
Item	Date of purchase	Value (in USD)
NOVA3D Bene5 resin printer	20/10/2023	$ 212.09
Silicone mats	24/12/2021	$ 23.52
Plastic trays	20/11/2021	$ 42.80
ANYCUBIC Wash&Cure 3	24/01/2023	$ 177.23
Total initial investment	‒	$ 455.65

**Consumables per unit**		
Item	Amount considered	Value (in USD)
Resin (3D Lab standard)	85.74 mL	$ 3.39
Isopropyl Alcohol	85.74 mL	$ 0.33
Nitrile gloves	Three pairs	$ 0.20
Power consumption	0.1 kWh	$ 0.01
Total consumable cost per unit	‒	$ 3.93

**Unit cost per number produced**		
For 10 units (including initial investment)		$ 49.50
For 30 units (including initial investment)		$ 19.12
For 100 units (including initial investment)		$ 8.49
Material-only cost per unit		$ 3.93

USD, United States Dollars.

Eleven participants performed dissection: five second-year residents, five third-year residents and one otology fellow.

Two participants had no previous experience, five were beginners who had performed tympanostomy tube placement and/or inlay tympanoplasty. Three more experienced participants had performed at least one previous mastoidectomy, and the otology fellow had previous experience with both mastoidectomy and cochlear implantation.

All of them completed a full mastoidectomy with either facial nerve exploration or canal wall down procedure. One student performed a simulated round window cochlear implant insertion using a mock-up electrode, because previous steps were completed very efficiently. The results of the dissection questionnaire are presented in Table 2 – training utility received the highest possible score from all participants. Likert scores of 1 and 2 are omitted, as no participant selected these values.

**Table 2:** Responses to quality assessment questionnaire.

Likert scale responses *Numbers 1 and 2 both had zero answers and were omitted.	3	4	5
Based on your current knowledge and experience, how do you evaluate the quality and detail of the bone anatomy encountered?	1 (9.1%)	3 (27.3%)	7 (63.6%)
Based on your current knowledge and experience how do you evaluate the soft tissue anatomy encountered: sigmoid sinus, dura mater, and facial nerve?	4 (36.4%)	5 (45.5%)	2 (18.2%)
Based on your current knowledge and experience how do you evaluate the bone dissection experience (tactile, visual, and auditory feedback).	1 (9.1%)	4 (36.4%)	6 (54.5%)
How do you perceive the safety of the synthetic model, when compared to a human temporal bone (fresh or fixed in formalin)?	1 (9.1%)	1 (9.1%)	9 (81.8%)
How do you rate the utility of this temporal bone model for ear surgery training?	0	0	11 (100%)

Likert scale responses according to participant experience	Average answer	Average (2^nd^ year)	Average (3^rd^ year)	Otology fellow
Based on your current knowledge and experience, how do you evaluate the quality and detail of the bone anatomy encountered?	4.55	4.20	5	4
Based on your current knowledge and experience how do you evaluate the soft tissue anatomy encountered: sigmoid sinus, dura mater, and facial nerve?	3.82	3.60	4.20	3
Based on your current knowledge and experience how do you evaluate the bone dissection experience (tactile, visual, and auditory feedback).	4.45	4.20	4.80	4
How do you perceive the safety of the synthetic model, when compared to a human temporal bone (fresh or fixed in formalin)?	4.73	4.40	5	5
How do you rate the utility of this temporal bone model for ear surgery training?	5	5	5	5

The Spearman's rank correlation results are shown in Table 3. We observed a moderate tendency for more experienced participants to rate anatomy higher than less experienced participants. Conversely, dissection feel showed a weak negative correlation with experience, and more experienced participants also tended to rate the safety of dissection less favorably. However, no correlation reached statistical significance (all p > 0.05); the closest was the rating of bony anatomy (p = 0.0938).

**Table 3:** Spearman’s ranked correlation (years of training vs. responses).

	*r*	p-Value
Based on your current knowledge and experience, how do you evaluate the quality and detail of the bone anatomy encountered?	0.5584	0.0938
Based on your current knowledge and experience how do you evaluate the soft tissue anatomy encountered: sigmoid sinus, dura mater, and facial nerve?	0.4639	0.1769
Based on your current knowledge and experience how do you evaluate the bone dissection experience (tactile, visual, and auditory feedback).	0.2018	0.5763
How do you perceive the safety of the synthetic model, when compared to a human temporal bone (fresh or fixed in formalin)?	0.3376	0.3402
How do you rate the utility of this temporal bone model for ear surgery training?	0.0	1.0

## Discussion

The initial motivation for this project was that, although several temporal bone models for surgical training have been published, production costs were seldom analyzed.^1^^,^^6^^,^^15^ More recently, two articles have addressed this issue.^16^^,^^17^ Lee et al.^17^ used a markedly different approach, employing a lattice structure inside the mastoid that allows students to dissect manually without drilling. Our intention was for dissection to simulate actual ear surgery using a microscope and surgical drill, similar to the approach of Nwosu et al.,^16^ who also analyzed the costs of model production. Our project achieved a lower per-unit cost of USD 3.93, compared with USD 6.40. The largest difference, however, was in the initial investment: USD 6,232.10 reported by Nwosu et al.,^16^ versus USD 455.65 in our study. This difference is explained by our use of consumer-grade printers. A model with low initial investment may be particularly well suited for adoption in a developing country such as ours, and is especially relevant given the steep learning curve of ear surgery, which requires repetitive training.^15^ The total cost of all equipment plus production of one hundred units remains below USD 1000, at USD 849.00. To our knowledge, this is the lowest-cost temporal bone training model reported in the literature to date.

Resin printers were chosen over Fused Deposition Modeling (FDM) printers (“filament printers”). FDM printers use thermoplastics and do not require additional curing or washing; however, they melt and deform under heat, such as the heat generated by drilling. They also produce a rougher surface finish and lower detail resolution, commonly above 0.1 mm (100 μm), compared with 50 μm or less for most resin printers. Because middle ear anatomy is small, intricate, and typically viewed under a microscope, high printer resolution is essential.

Potential for training purposes was perceived as high. No responses attributed scores 1 or 2, and most chose 4 or 5 to every question. Training utility received a perfect score of 5 from all participants. That was an initial concern, as low cost was pursued and hence quality could be perceived as lacking.

Bone anatomy was rated higher than soft tissue anatomy, a consistent finding in comparable models in literature and a known limitation of resin printing technology, which is best suited to rigid materials.^18^ Despite that fact, our model has a feature in the preparation of the Fallopian canal for silicone injection, a solution that allows for realistic facial nerve exploration and transposition.

All participants gave the model the highest rating (5) for training utility, in contrast to the more variable responses regarding anatomical quality and dissection feel. While the model may be particularly useful for beginners, this rating may also reflect the limited opportunities for temporal bone dissection in their residency program, suggesting that the model fills an important gap in their training.

The Likert scale used is a similar method of assessing model quality to other exploratory studies in 3D printed temporal bones.^9^^,^^14^ Most studies in this field have a small number of participants, comprised usually of students and staff of the programs or universities in which they were developed. We aimed to replicate a similar methodology, but the true aim was to describe the costs and materials involved, which is not commonly described.^9^^,^^16^

Safety is a recognized concern with resin printing because of the toxicity of commercial resins. Printing requires a well-ventilated space and nitrile gloves to prevent skin irritation.^19^ Dissection was performed with the same precautions used for human bone, including a surgical mask, gloves, and gown or scrubs. Protective glasses were used during cortical dissection and removed when dissection was performed under the microscope. A previous study by Freiser et al.^20^ found these models to be safe for dissection and compliant with United States occupational safety regulations. In the present study, participants did not perceive the model as unsafe.

None of the correlations reached statistical significance (all p > 0.05). Nonetheless, participants' overall perception of the model was positive, with most rating its anatomical fidelity highly and more experienced participants tending to give higher ratings.

A primary limitation of our validation process is the small sample size (n = 11), which restricts the statistical power of our comparative analysis between novice and experienced surgeons. While a larger cohort would undoubtedly yield a more robust assessment of the model's educational impact, this sample size reflects the current state of surgical simulation literature, where initial evaluations of novel 3D-printed prototypes frequently rely on similarly limited participant pools.^14^^,^^18^ Furthermore, it is essential to emphasize that the primary scope of this manuscript is to demonstrate the feasibility, reproducible manufacturing workflow, and cost analysis of an ultra-low-cost model. Even though the production process requires more than 24 -hs from print to ready-for-use, owing to post-processing, the simplicity of the process allows residents themselves to produce these temporal bone models once they are integrated into the residency program.

## Conclusion

A low-cost temporal bone model was developed that enables repeated practice to address the steep learning curve of ear surgery. The reduced financial burden may broaden access to temporal bone dissection training, complementing the use of cadaveric specimens where available and strengthening ear surgery education overall. The model combines a reproducible manufacturing workflow with an exceptionally low production cost while maintaining high perceived educational value.

## Data availability

The authors declare that all data are available in repository.

## Declaration of competing interest

The authors declare no conflicts of interest.

## References

[1] Frithioff A., Frendø M., Pedersen D.B., Sørensen M.S., Wuyts Andersen S.A. (2021). 3D-printed models for temporal bone surgical training: a systematic review. Otolaryngol Head Neck Surg.

[2] Frithioff A., Frendø M., Weiss K. (2021). Effect of 3D-printed models on cadaveric dissection in temporal bone training. OTO Open.

[3] Naik S.M., Naik M.S., Bains N.K. (2014). Cadaveric temporal bone dissection: is it obsolete today?. Int Arch Otorhinolaryngol.

[4] George A.P., De R. (2010). Review of temporal bone dissection teaching: how it was, is and will be. J Laryngol Otol.

[5] Kryukov A.I., Garov E.V., Zelenkova V.N. (2020). [Artificial temporal bone]. Vestn Otorinolaringol.

[6] Chen G., Jiang M., Coles-Black J., Mansour K., Chuen J., Amott D. (2020). Three-dimensional printing as a tool in otolaryngology training: a systematic review. J Laryngol Otol.

[7] admin. Adquira suas peças. Otobone. [Accessed 9 February 2025]. https://otobone.com.br/en/adquira-otobone/.

[8] PHACON Temporal Bone Patient „Schmidt “– 55 Jahre – normale Anatomie – Phacon. [Accessed 8 May 2022]. https://phacon.de/patienten/patient-schmidt/.

[9] Teoh Q., Chngh J.S., Tow G.R. (2026). 3D-printed temporal bone models versus cadaveric bone for mastoidectomy training: a feasibility and cost analysis. Eur Arch Otorhinolaryngol.

[10] Sieber D., Erfurt P., John S. (2019). The OpenEar library of 3D models of the human temporal bone based on computed tomography and micro-slicing. Sci Data.

[11] blender.org ‒ Home of the Blender project ‒ Free and Open 3D Creation Software. [Accessed 31 January 2025]. https://www.blender.org/.

[12] Download the latest version of Lychee Slicer for SLA/Resin 3D Printers. Mango 3D. [Accessed 8 May 2022]. https://mango3d.io/downloads/.

[13] Takahashi K., Morita Y., Ohshima S. (2017). Creating an optimal 3D printed model for temporal bone dissection training. Ann Otol Rhinol Laryngol.

[14] Ock J., Choi Y., Lee D.G., Chung J.W., Kim N. (2024). Educational simulator for mastoidectomy considering mechanical properties using 3D printing and its usability evaluation. Sci Rep.

[15] Kashikar T.S., Kerwin T.F., Moberly A.C., Wiet G.J. (2019). A review of simulation applications in temporal bone surgery. Laryngoscope Investig Otolaryngol.

[16] Nwosu O.I., Quesnel A.M., Crowson M.G., Gray S.T. (2025). Considering the costs: resin 3D printing for a temporal bone dissection course. Otolaryngol Head Neck Surg.

[17] Lee S.Y., Jeong B., Lee W., Park M.K. (2024). Feasibility of easy to use and inexpensive three-dimensional printed educational model of temporal bone: practiced without drilling. J Med Educ Curric Dev.

[18] Iannella G., Pace A., Greco A. (2025). The SAPIENS 3D-printed temporal bone model: a real tool for advanced otologic surgery education. Eur Arch Otorhinolaryngol.

[19] Shahrubudin N., Lee T.C., Ramlan R. (2019). An overview on 3D printing technology: technological, materials, and applications. Procedia Manuf.

[20] Freiser M.E., Ghodadra A., Hart L., Griffith C., Jabbour N. (2018). Safety of drilling 3-dimensional-printed temporal bones. JAMA Otolaryngol Head Neck Surg.

